# Role of P2 Receptors as Modulators of Rat Eosinophil Recruitment in Allergic Inflammation

**DOI:** 10.1371/journal.pone.0145392

**Published:** 2016-01-19

**Authors:** Anael Viana Pinto Alberto, Robson Xavier Faria, Joao Ricardo Lacerda de Menezes, Andrea Surrage, Natasha Cristina da Rocha, Leonardo Gomes Braga Ferreira, Valber da Silva Frutuoso, Marco Aurélio Martins, Luiz Anastácio Alves

**Affiliations:** 1 Laboratório de Comunicação Celular, Instituto Oswaldo Cruz, Fundação Oswaldo Cruz, Rio de Janeiro, Brasil; 2 Laboratório de Inflamação, Instituto Oswaldo Cruz, Fundação Oswaldo Cruz, Rio de Janeiro, Brasil; 3 Laboratório de Neuroanatomia Celular, Instituto de Ciências Biomédicas, Universidade Federal do Rio de Janeiro, Rio de Janeiro, Brasil; 4 Laboratório de Imunofarmacologia, Instituto Oswaldo Cruz, Fundação Oswaldo Cruz, Rio de Janeiro, Brasil; Universidade Federal do Rio de Janeiro, BRAZIL

## Abstract

ATP and other nucleotides are released from cells through regulated pathways or following the loss of plasma membrane integrity. Once outside the cell, these compounds can activate P2 receptors: P2X ionotropic receptors and G protein-coupled P2Y receptors. Eosinophils represent major effector cells in the allergic inflammatory response and they are, in fact, associated with several physiological and pathological processes. Here we investigate the expression of P2 receptors and roles of those receptors in murine eosinophils. In this context, our first step was to investigate the expression and functionality of the P2X receptors by patch clamping, our results showed a potency ranking order of ATP>ATPγS> 2meSATP> ADP> αβmeATP> βγmeATP>BzATP> UTP> UDP>cAMP. This data suggest the presence of P2X1, P2X2 and P2X7. Next we evaluate by microfluorimetry the expression of P2Y receptors, our results based in the ranking order of potency (UTP>ATPγS> ATP > UDP> ADP >2meSATP > αβmeATP) suggests the presence of P2Y2, P2Y4, P2Y6 and P2Y11. Moreover, we confirmed our findings by immunofluorescence assays. We also did chemotaxis assays to verify whether nucleotides could induce migration. After 1 or 2 hours of incubation, ATP increased migration of eosinophils, as well as ATPγS, a less hydrolysable analogue of ATP, while suramin a P2 blocker abolished migration. In keeping with this idea, we tested whether these receptors are implicated in the migration of eosinophils to an inflammation site in vivo, using a model of rat allergic pleurisy. In fact, migration of eosinophils has increased when ATP or ATPγS were applied in the pleural cavity, and once more suramin blocked this effect. We have demonstrated that rat eosinophils express P2X and P2Y receptors. In addition, the activation of P2 receptors can increase migration of eosinophils in vitro and in vivo, an effect blocked by suramin.

## Introduction

Extracellular nucleotides have been recognized as important mediators in many systems, where they trigger different responses via activation of plasma membrane receptors known as P2 receptors[1]. The different subclasses of P2 receptors have been identified on a wide variety of cell types: muscle, endothelial, endocrine and others; including cells of the immune system: lymphocytes, neutrophils, macrophages, mast cells[2]; as well as eosinophils [3,4].

P2 receptors are divided in two families: P2Y and P2X. P2X receptors are identified as selective channels for monovalent and divalent cations which are directly activated by extracellular ATP and do not require the hydrolysis of the nucleotide or generation of intracellular secondary messengers [5,6]. Stimulation of P2X receptors causes a Ca^2+^ and Na^2+^ influx according to electrochemical gradient and the accompanying plasma membrane depolarization [7]. Seven different monomers of P2X have been cloned in mammals and named P2X_1_–P2X_7_[8]. Whereas P2Y receptors have seven membrane spanning segments and are G-protein-coupled receptors. Their activation triggers generation of inositol 1,4,5-trisphosphate and release of Ca2+ from the intracellular stores. Of the P2Y subtypes, 8 have been cloned in mammals (P2Y_1_, P2Y_2_, P2Y_4_, P2Y_6_, P2Y_11_, P2Y_12_, P2Y_13_, and P2Y_14_) [9].

Human eosinophils have been shown to express P2X_1_, P2X_4_, P2X_5_ and P2Y_1_, P2Y_2_, P2Y_4_, P2Y_6_ and P2Y_11_, and when primed with IFN-α these cells can also express the receptor P2X_7_ [4]. Human P2Y_12_ receptor also was described in eosinophils by Neves and colleagues [10]. The activation of these receptors by ATP can trigger actin reorganization, increase of intracellular calcium, CD-11b up-regulation, oxygen radical production and chemotaxis [3,11,12]. The activation of P2 receptors by extracellular nucleotides also induces release of IL-8 and eosinophil cationic protein which is blocked by the antagonist of P2X_7_ receptor KN-62 and by pertussis toxin [13]. Thus, indicating a participation of different purinoceptors and signaling pathways in the regulation of cell responses in eosinophils.

Traditionally associated with parasitic infections or allergic manifestations, eosinophils [14,15], play a key role in several diseases including asthma [16,17], allergy [18] and infections by helminthes [19]. Physiological functions of eosinophils are related to their ability to produce, store and release many biologically active molecules. Data suggest that eosinophils also express MHC class 2, which suggests that eosinophils could act as antigen presenting cells [20,21]. As effector cells, eosinophils can have roles that are both beneficial and detrimental to the host [22].

As eosinophils are involved in asthma, the characterization of P2 receptors and the effects of these receptors in eosinophils are essential for understanding the biology of eosinophils in health and disease. Animal models of diverse species are used to study the pathophysiology of the asthma. Among them, rats exhibit a large number of characteristics found in airway allergy and allergic asthma that are similar humans symptoms [23]. In comparison to mouse model, rat models produce early- and late- stage asthmatic reaction and airway hyperresponsiveness [24,25]. Based on this, we investigated what receptors were expressed and the role of these P2 receptors in the rat eosinophil migration in vitro and in vivo.

## Materials and Methods

### Reagents

All chemicals were purchased from Sigma Chemical Co. (St. Louis, MO, USA), including ATP, UTP, ADP, UDP, αβ-meATP, ATPγS, 2meATP, Adenosine, Suramin, RPMI 1640 culture medium and Adenosine. All antibodies were polyclonal and purchased from Alomone Labs, Israel. All selective P2Y receptor used were obtained from Tocris Bioscience, Bristol, United Kingdom. The reagents were stored according manufacturer’s instructions.

### Animals

Male Wistar rats weighing 200 to 300 g (4–5 weeks) were obtained from CECAL/FIOCRUZ. All animal procedures were performed with the approval of the ethics committee–Animal Ethic Committee number obtained from Fiocruz: LW23/09.

### Isolation of eosinophils

Eosinophils were obtained from peritoneal lavage with RPMI and then treated on a two-layer Percoll gradient (56% and 72%; Gärtner, 1980). Alternatively, metrizamide discontinuous gradient centrifugation (18–24%) was performed [26]. The cells between 56–72% percollor 20–24% metrizamide were collected. In both cases, the eosinophils purity was≥90% as evaluated by a differential counter stained by May-Grunwald-Giemsa.

### Electrophysiological measurements

Cell attached and whole cell patch clamping were performed at 37°C using an Axopatch-1Damplifier (Axon Instruments, San Mateo, U.S.A). Cells were transferred to a chamber mounted in a microscope stage. Patch pipettes (with 1.2 mm filament) were pulled from IBBL borosilicate glass capillaries with resistance between 6 to 10 MΩ (World Precision Instruments, New Haven, U.S.A).

A high resistance seal (1–10 GΩ) was established by gentle suction, after which the cell membrane was disrupted beneath the tip of the electrode by additional suction. Leakage was found to be negligible (currents in the absence of agonist were <0.1% of maximal agonist-induced currents). Current and membrane conductance was recorded when it returned within 1–5% of control values after agonist application, verifying that increase was not due to cell lysis with loss of seal.

Series resistance was 6–10 MΩ for all experiments. Currents were compensated by 85% with the exception of currents <400 pA. Trials in which the series resistance increased substantially during measurement were discarded. Cell capacitance (9.2±3.2 pF, n = 82) was measured by applying a 20 mV hyperpolarizing pulse from a holding potential of –20 mV; capacitive transient was then integrated, and divided by the amplitude of the voltage step (20 mV).

Voltage clamp protocols applied holding potentials of -60 mV. Currents were filtered with a corner frequency of 5 kHz (8-pole Bessel filter), digitized at 20–50 kHz using a Digidata 1320 interface (Axon Instruments, Palo Alto, CA, U.S.A), and acquired in a personal computer using Axoscope software (Molecular Devices, California, USA).

### Saline solutions for electrophysiology

Different saline solutions were used in the pipette or in the bath depending on the protocol: Solution A (in mM): 150 NaCl, 5 KCl, 1 MgCl_2,_ 1 CaCl_2_ and 10 HEPES, pH 7.4; solution B (in mM): 150 KCl, 5 NaCl, 1 MgCl_2_, 1 CaCl_2_, 10 HEPES, and 0.1 EGTA, pH 7.4.

### Membrane potential control

Solution A was used inside the pipette and solution B in the bath to record single channel currents. 1) Solution A was placed inside the pipette warranted more physiological ion gradients across the patch to more efficiently study the nucleotide activated phenomena solution. Solution B was placed in the bath which should have completely depolarize the cell membrane (~0mV) to assure, that the real value of the voltage potential across the patch was virtually the same as the nominal holding potential applied.

### Drug application

Drugs were mostly administered by using an automatic micropipette with variable volume (Gilson, France). The micropipette tip was positioned inside the bath solution near the cell to record the base line. After few seconds the durg was delivered Some experiments were performed under automatized perfusion (RC-24 chamber, Warner Instrument Corporation, Handem, USA) to confirm the data obtained through micropipette application. All drugs were dissolved in saline solution immediately before use.

### Intracellular calcium measurement

Eosinophils (1 x 10^5^cells/mL) were incubated with 2.5 μM Fura-2/AM for 50 minutes at room temperature. Extracellular Fura-2 was washed and then removed. Initially, the experiments were done using a photomultiplier from (Photon Technology, Princeton, NJ) in a modified 3-compartment superfusion chamber with the bottom formed by the coverslip (27). Eosinophils were chosen and excited at 340- and 380-nm wavelengths and measured the ratio of emission at 510 nm (340 nm/380 nm). Thus, the approximate intracellular calcium concentration was calculated from ratio of fluorescence at each wavelength excitation [27]. Eosinophils were perfused with standard saline at room temperature and drugs were applied in the initial mixing compartment, reaching the cells at a final concentration depicted in Fig legends.

Some experiments were performed, using a FlexStation III plate reader (Molecular Devices). For these assays, cells were loaded with 2 μM Fluo-4AM calcium indicator dye (Molecular Probes), with 0,02% pluronic acid F-127 (Molecular Probes), 250 μM sulfinpyrazone (Sigma) in Dmem F/12 (Sigma) with 10% FCS. Loading was performed for 60 minutes at 37°C. The medium was removed and wells were washed twice and replaced with Krebs buffer n (132 mM NaCl, 4 mM KCl, 1.4 mM MgCl2, 2.5 mM CaCl2, 6 mM glucose, 10 mM HEPES, pH 7.4). Cells were maintained for 5 minutes at 37°C before measurements started and P2 receptor agonists were injected automatically. Fluorescence was measured using a 494 nm excitation and 525 nm emission (cut off 515 nm) and fluorescence measurements were taken every 2 seconds.

### Chemotaxis

The migration experiments were performed in a transwell (Sigma-Aldrich Co.; St. Louis,MO, USA), using a 3-μm membrane filter. The bottom wells were loaded with RPMI, with and without agonists. Eosinophils (10^5^ cells/ml) were added into the upper wells. The chamber was incubated at 37°C for 1 or 2 hours in the presence of 5% CO_2_ [26].

### Immunofluorescence assays

Cells were distributed onto glass blades fitted within a Cytospin 2, fixed with 4% paraformaldehyde (in PBS puffer, pH 7.4) for 5 min at 25°C and washed three times for five minutes in the same buffer. Fixed cells were then resuspended with PBS, pH 7.4, Triton-X100 0.1% and goat serum (blocking buffer). All antibodies were used at dilution of 1:200 with exception for P2X7 receptor antibody, 1:100. Isotype control antibody (IgG) was produced by rabbits. Secondary antibody was used at dilution of 1:400 (Invitrogen,California, USA).

Primary antibodies were incubated overnight at 4°C, followed by three additional washings with PBS. Secondary labeling was performed with anti-rabbit antibody conjugated with Alexa 546 for two hours at room temperature. After three washes with PBS we add mounting media, and cells were viewed on a Nikon inverted microscope TE2000 using a 40X objective lens, equipped with an image acquisition system and analyzed “Image pro 4” software (Media Cybernetics, Bethesda, MD).

### Pleurisy assays

Rats were sensitized by sub cutaneous injection of 0.2 ml of an ovalbumin (5mg/50mg)/Al(OH)^3^ saline solution [27]. Fourteen days later, the animals were submitted to an intrathoracic injection of ovalbumin (100 μL, 12μg/cavity). The rats were treated with agonists or antagonists of P2 receptors 1 hour before the challenge. Twenty-four hours later, the rats were killed and thoracic cavity was rinsed with 3 mL of sterile saline. Pleural washing was collected and total cell counts were obtained with a Neubauer chamber in an optical microscope. Differential eosinophils counts were performed in cytocentrifuged smears stained with May-Grunwald-Giemsa dye under oil immersion objective lens (100x). All experiments were made in 3 independent days with 6 animals per group.

### Statistical analysis

Unless otherwise stated, data are expressed as the mean±SEM. Analysis of variance (ANOVA) was used to compare experimental groups to control values. When the global test of differences were significant at the 5% level, pairwise test of differences between groups were applied (Bonferroni’s Multiple Comparison Test).

## Results

### Ionic currents elicited by the activation of P2X receptors

**Fig 1:**
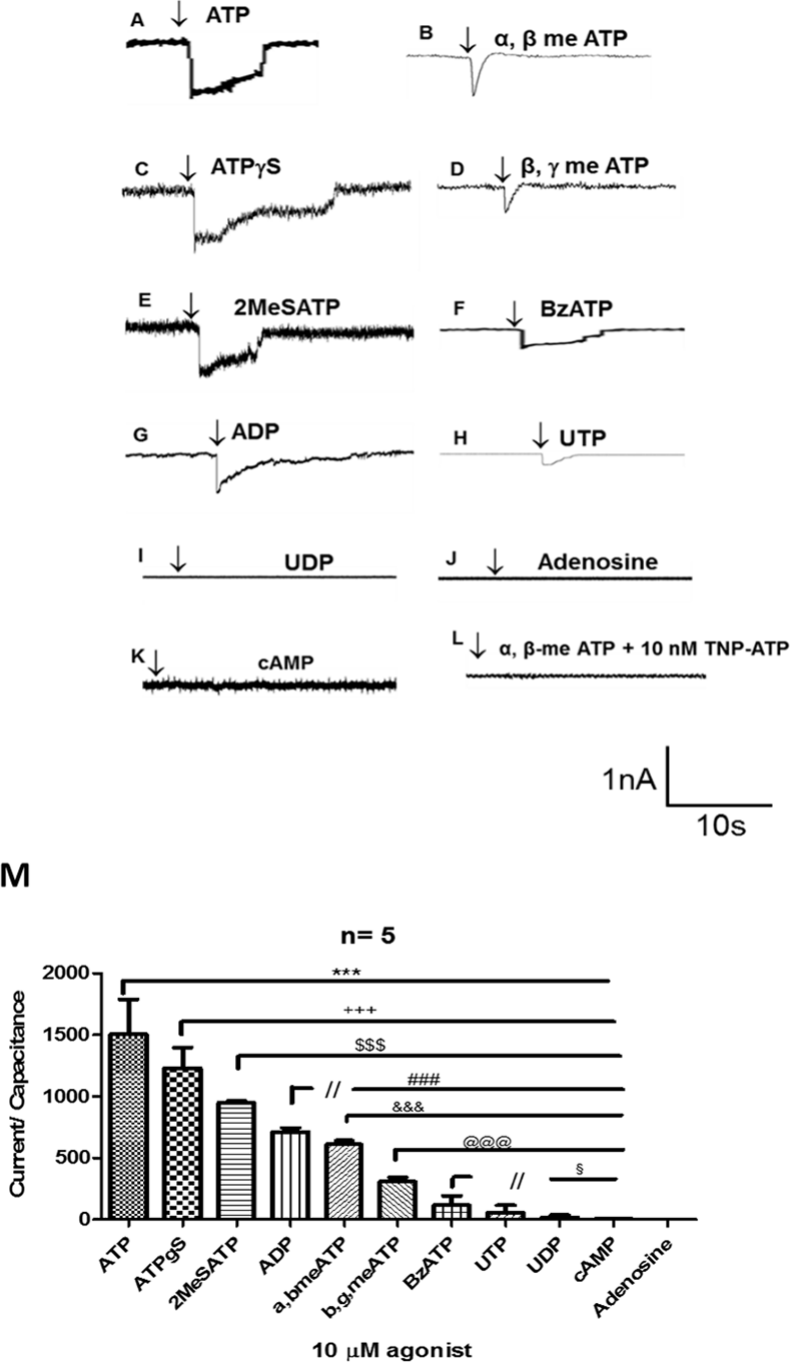
Currents elicited by the activation of P2X receptors in eosinophils. All records were obtained at 37°C. All agonists were applied at concentration of 10 μM and we maintained the Pipette holding of (Vhold = -60 mV). (1A-L) Patch clamping whole cell recordings after application of P2 receptor agonists. Arrows indicate the point of agonist application. (1M) Bar graph representing the quantitative relation of the ionic currents amplitude normalized by cell capacitance, after agonist activation of P2X receptors (n = 5 independent experiments). *** p< 0.01 when ATP was compared to other agonists; +++ p<0.01 when ATPγS was compared to other agonists; $ $ $ p<0.01 when 2MeSATP was compared to other agonists; ### p<0.01 when ADP was compared to other agonists; &&&p<0.01 when α,β-meATP was compared to other agonists; @@@ p<0.01 when β,γ-meATP was compared to other agonists; § p<0.05 when BzATP was compared to other agonists.

We first accessed the pharmacological profile of P2X receptors in rat eosinophils performing whole cell patch clamping recordings after application of different agonists. We compared the pharmacological potency of the agonists applying 10 μM of each agonist. As demonstrated in Fig 1, ATP elicited the higher current (1503,8 ± 105,7 pA/pF; Fig 1A). Fig 1A–1L show the recordings elicited by application of diverse P2R agonists (arrow). Surprisingly, ADP (710,7 ± 26,44 pA/pF; Fig 1G) and UTP (44,3 ± 3,7 pA/pF; Fig 1H), in general P2Y agonists, elicited small currents when compared to ATP response. Adenosine (Fig 1J), UDP (Fig 1I) and the cell permeable AMP (cAMP) (Fig 1K) did not have any effect as expected. All artificial ATP analogues promoted ionic current activation (Fig 1B–1F).

A bar graph (Fig 1M) shows the analysis of five different experiments and all agonists were found to be statistically different of each other with exception of BzATP, which was found to be similar to UTP. According to the relative potency order of the agonists [28] and the order established through Fig 1M, our data suggest the essential functional activity of the P2X_2_ receptor based on rank published by Burnstock and Knight [29]. Moreover, other several subtypes of P2X receptors may exhibit functional activity. 2-MeSATP may activate P2X_3_ and poorly P2X_1_ and P2X_7_ receptors. The weak BzATP response reinforces the reduced P2X_7_ receptor activity observed previously [30]. Additionally, the temporal profile of the ionic currents induced by αβmeATP and βγmeATP is one criteria used to characterized P2X_1_ and P2X_3_ receptors. In consequence, the closed time of the recordings showed a τ = 386,2 ± 20,1ms for αβmeATP, 409,6 ± 18,8 ms for βγmeATP, possibly indicating functional P2X_1_ and/or P2X_3_ receptors, as these parameters are very similar to the described in the literature for both receptors or heteromeric channels formed by them [31,32]. P2X_1_ receptor involvement was confirmed by treatment with TNP-ATP a P2X_1_ receptor antagonist in nanomolar range. As observed in the Fig 1L, TNP-ATP (10 nM) inhibited αβmeATP induced current.

### Analysis of calcium transients activated by nucleotides

**Fig 2:**
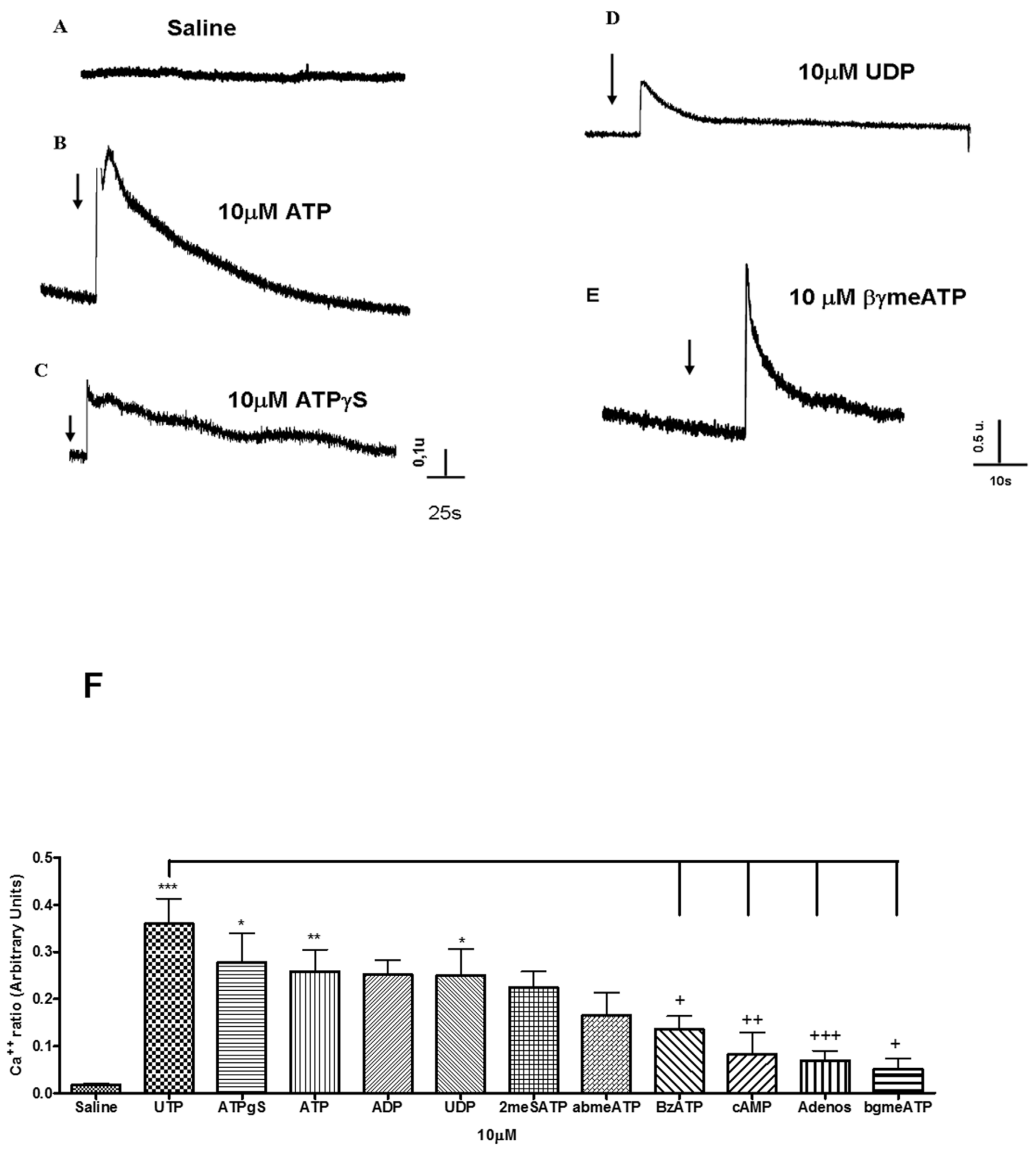
Analysis of calcium transients activated by nucleotides. Rat eosinophils were incubated at 37°C. 2A- represents the baseline. 2B- Final concentration of 10 μM ATP application, after a period of 2 minutes in saline. The arrows represents the moment of agonist application.2C- 10 μM ATPγS application. 2D- 10 μM UDP and 2E- 10 μM β,γ-meATP. All recordings were performed at distinct cells. (2F) Bar graph representation of 3 independent experiments. *** p<0.01 when baseline (saline) was compared to UTP treatment. ** or * p<0.05 when there was statistical significance between saline and some agonist listed. +++ p<0.01, ++ or + p<0.05 when there was statistical significance between UTP and some agonist listed.

Cells were loaded with FURA2-AM and the mobilization of intracellular calcium was evaluated by a microfluorimeter. The application of the agonists activated both P2X and P2Y receptors. First component is the fast early peak suggesting a P2X receptor activation and a second component, slower and sustained, indicative of P2Y receptors activation. To establish P2Y receptor participation, we considered only the second component. A signal three times superior to the media of baseline values fluctuation was considered as a positive response. Fig 2A represents the baseline before the treatment. All agonists were tested in a concentration of 10 μM. We considered as stimulation, when the agonist promoted a calcium signal, at least, two times superior to baseline signal. Fig 2B–2E is representative of ATP, ATPγS, UDP and βγmeATP activity respectively. UTP was found to be the most potent agonist tested, ATPγS, ATP and UDP promoted a similar calcium response to UTP. Other agonist did not generate calcium signal different from baseline levels. Based on potency rank [29,32] compared to rank observed at Fig 2F, the P2Y2 are the key P2Y receptors involved. Although, the involvement of P2Y4 receptor could not be ruled out, since UTP response was higher than ATP effect, this difference was not statistically significant. UDP response indicates P2Y4 receptors activation and essentially P2Y6 receptor involvement. ATPγS induced calcium signal is an indicative of P2Y11 receptor activation, but it may also activate P2X4 receptor and poorly the P2X7 receptor.

### Immunolabeling of P2 receptors expressed by eosinophils

**Fig 3:**
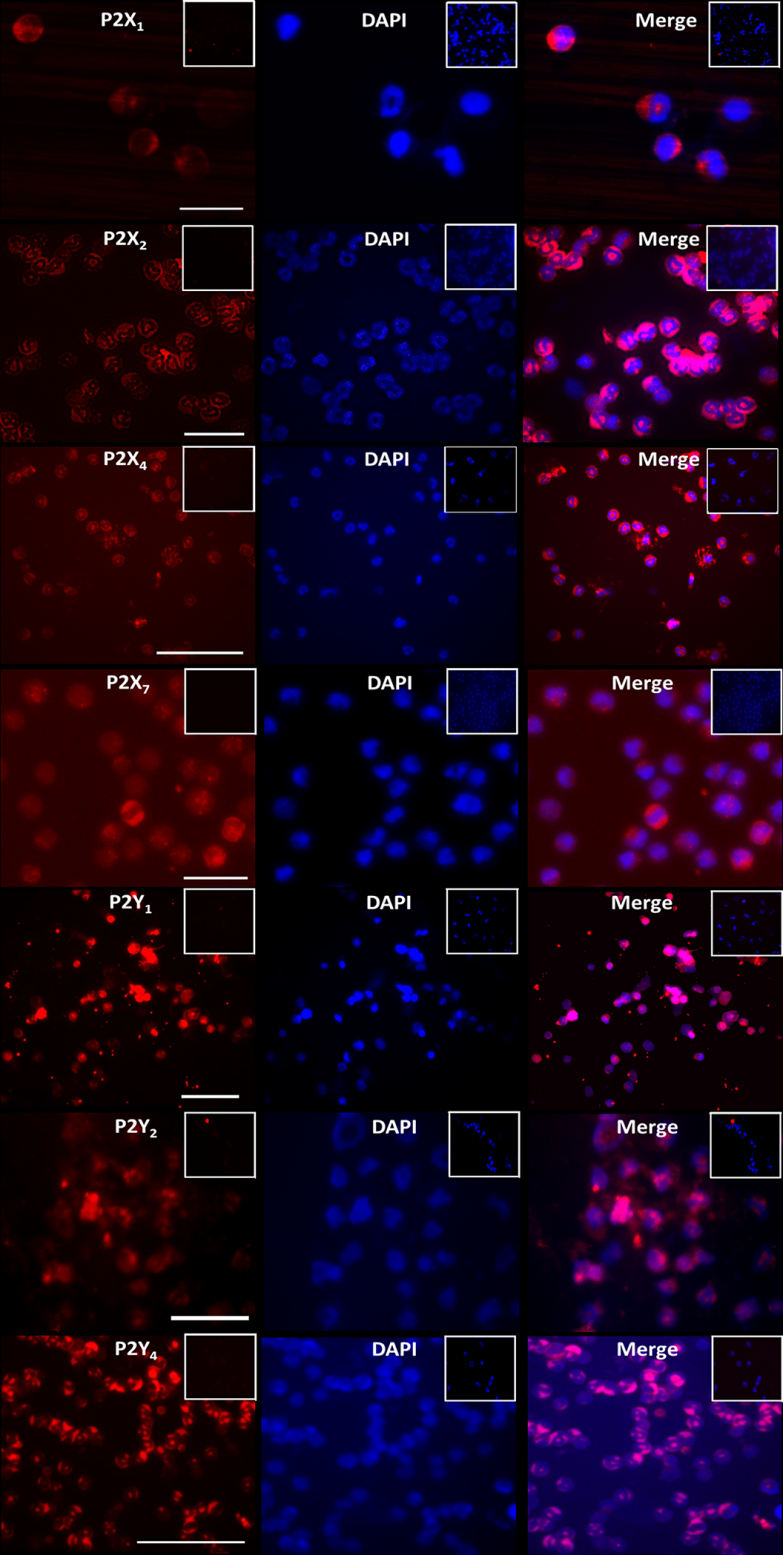
Immunofluorescence assays for P2 receptors in eosinophils. Cells were fixed in a quantity of 10^5^ cells per experiment. The immunofluorescence was done against P2XR and P2YR, with rabbit primary antibodies (Alomone Labs), and then a secondary antibody conjugated to Alexa red 546 was added to the preparation and visualized in the fluorescence microscope. We had positive reaction for P2X1, P2X_2_, P2X_4_, P2X_7_ and P2Y_1_, P2Y_2_, P2Y_4_.These images were obtained with objective of 40X.

In order to verify whether the expression of P2 receptors corroborate with our findings in functional assays, we performed immunofluorescence analysis on eosinophils and demonstrated the expression of P2X_1_, P2X_2_, P2X_4_, P2X_7_ and P2Y_1_, P2Y_2_, P2Y_4_ (Fig 3). Our results suggest a diffuse intracellular receptor localization in all subtypes tested with, in some cases, a denser area of receptors close to the plasmatic membrane, as observed to P2X1, P2X4 (Fig 3A) and P2Y1 and P2Y2 (Fig 3B). The P2X7 receptor shows only a low number of events and this indicates a poor expression of P2X7 receptor at plasmatic membrane (Fig 3A). P2X2, P2Y1 and P2Y4 receptors exhibited a prominent intracellular localization.

### Chemotaxis induced by P2 receptors

**Fig 4:**
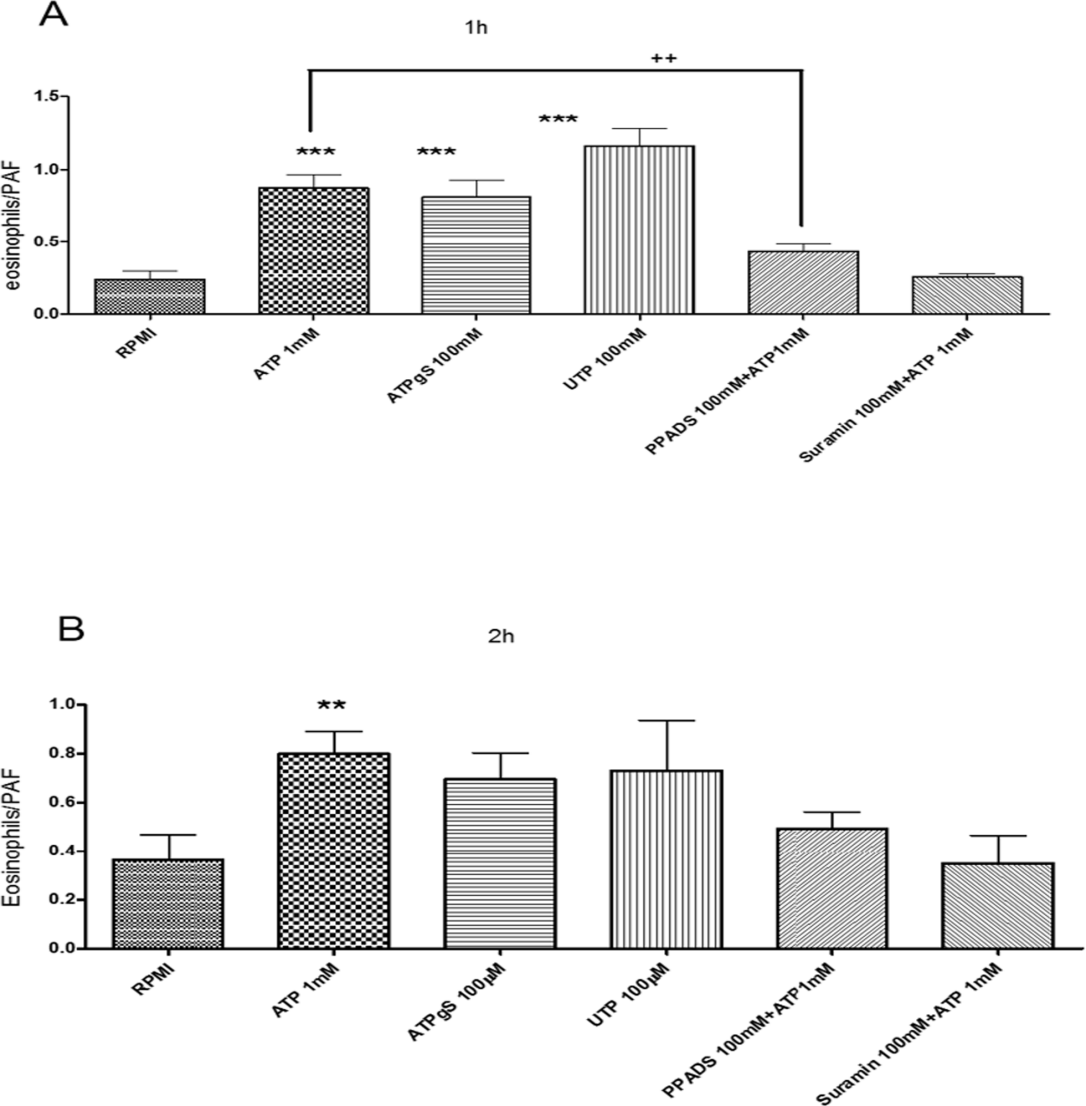
Assessment of rat eosinophil chemotaxis induced by nucleotides using transwell chamber. After 1h or 2h of incubation the cells that migrated were counted by hemocytometer. PAF was used as a positive control. Bar graph were normalized to PAF response. *** p<0.05 when treated cells were obtained statically significance in comparison to no treated cells (RPMI); ++ p<0.05 when ATP treatment was significant in relation to Suramin treatment. Results were performed in at least 3 independent days.

To evaluate a possible role of the purinergic receptors in eosinophil chemotaxis, as described by various cellular types such as macrophages, neutrophils, dendritic cells and human eosinophils [33,34], we performed a transwell migration assay of eosinophils toward a gradient of P2 receptors agonists. All agonists increased chemotaxis after incubation for 1h (Fig 4A) or 2h (Fig 4B), UTP was equipotent in relation to ATP and ATPγS in both cases, which suggests a main role of P2Y_2_ [32]_._ Based on UTP effect, P2Y_4_ and P2Y_6_ receptors may participate in eosinophils chemotaxis. ATPγS agonist activates P2Y_11_ receptor (Fig 4). ATP-induced chemotaxis was inhibited by the general P2 receptor antagonists Suramin and PPADS (Fig 4A and 4B). PPADS is known to exhibit a higher selectivity to P2X receptors, but it may also act poorly on P2Y_4_ and P2Y_6_ receptors [25]. Suramin also may poorly inhibit P2Y_4_ and P2Y_6_ receptor function [8,32].

Respective to P2X receptor participation, the similar response between ATP and ATPγS indicates a P2X_2_ receptor effect. However, a P2X_4_ receptor activation may also be present (Fig 4A). The ATP induced chemotaxis inhibition by PPADS and Suramin reinforces P2X_2_ receptor action (Fig 4A).

### Allergic pleurisy

**Fig 5:**
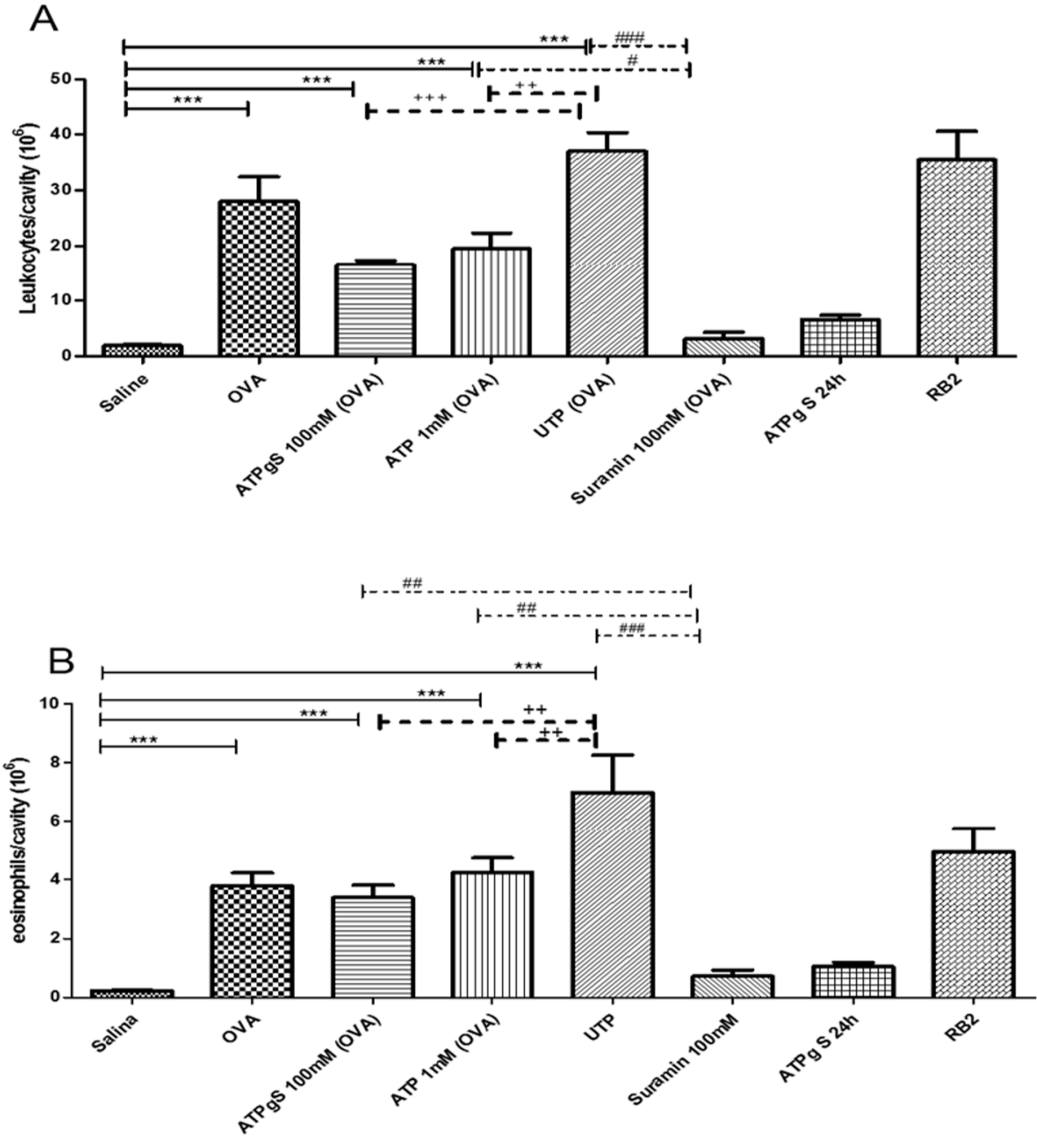
Modulation of migration by nucleotides in a pleurisy model. Rats were sensitized with OVA in the first day. On day 14 all animals were treated or not with agonists and antagonists of P2R, and after 1h animals were of challenged. After 24h, cells in the pleural cavity was collected and counted for total cells (Fig 5A) and eosinophils (Fig 5B). *** p<0. 05 when saline group was compared to other treatments; +++ p<0.01 when UTP group was compared to ATPγS group; ++ p<0.05 when UTP group was compared to ATP group; # p<0.05 when ATP group was compared to Suramin group; ### p<0.01 when UTP group was compared to Suramin group.This experiment was realized for 3 times with 6 animals per group.

To confirm our in vitro findings in a more pathological/physiological condition, we used the allergen-induced pleurisy model in sensitized rats. OVA induced inflammatory responses augmented the total number of leukocytes (Fig 5A) and eosinophils quantity (Fig 5B) compared to saline treated animals. We found that Suramin, a general blocker of P2 receptors, was able to block OVA-induced migration of leukocytes (Fig 5A) and eosinophils (Fig 5B) into the pleural cavity. This data points to P2 receptors participation in airways allergic response. Additionally, the addition of ATP, ATPγS and UTP increased total cells per cavity (Fig 5A and 5B) after sensitization with OVA. Among P2 receptor agonists, UTP (OVA) was the most potent to recruit total leukocytes and eosinophils (Fig 5A and 5B). ATP (OVA) and ATPγS (OVA) promoted equipotent recruitment of leukocytes and eosinophils (Fig 5A and 5B). UTP (OVA) and ATP (OVA) stimulus were inhibited by Suramin treatment, in contrast to ATPγS (OVA) stimulation (Fig 5A and 5B). Curiously, Reactive Blue 2 (RB2) treatment did not impair leukocytes and eosinophils augmentation in rats sensitized by OVA.

In accordance to agonist responses, UTP-induced cellular increase was higher than ATP response indicating the involvement of P2Y_4_, P2Y_6_ and P2Y_2_ receptor (Fig 5A and 5B). Suramin inhibited OVA, UTP (OVA) and ATP (OVA) effect, in contrast to RB2. Based on pharmacological rank to antagonist [29], among three P2 receptor with hypothetical participation listed above, only P2Y_2_ receptor is sensitive to Suramin and insensitive to RB2. In consequence, P2Y_2_ receptor is probably the key receptor actuating on allergen-induced pleurisy model.

### Chemotaxis and calcium analyses induced by selective P2Y receptors

To identify which P2Y receptor plays the major role in vitro eosinophils chemotaxis and in vivo pleurisy, we used MRS 2768, an agonist selective to hP2Y2 receptor (cat no. 3884); 2-S-UTP, hP2Y2 agonist with EC_50_ value of 0.035 nM, EC_50_ value of 0.035 nM to hP2Y4 receptor and EC_50_ value of 1.5 μM to hP2Y6 receptor (cat no. 3280); and MRS 4062 (cat no. 4261), hP2Y4 agonist with EC_50_ value of 23 nM, EC_50_ value of 640 nM to P2Y2 receptor and EC_50_ value of 740 nM to P2Y6 receptor.

**Fig 6:**
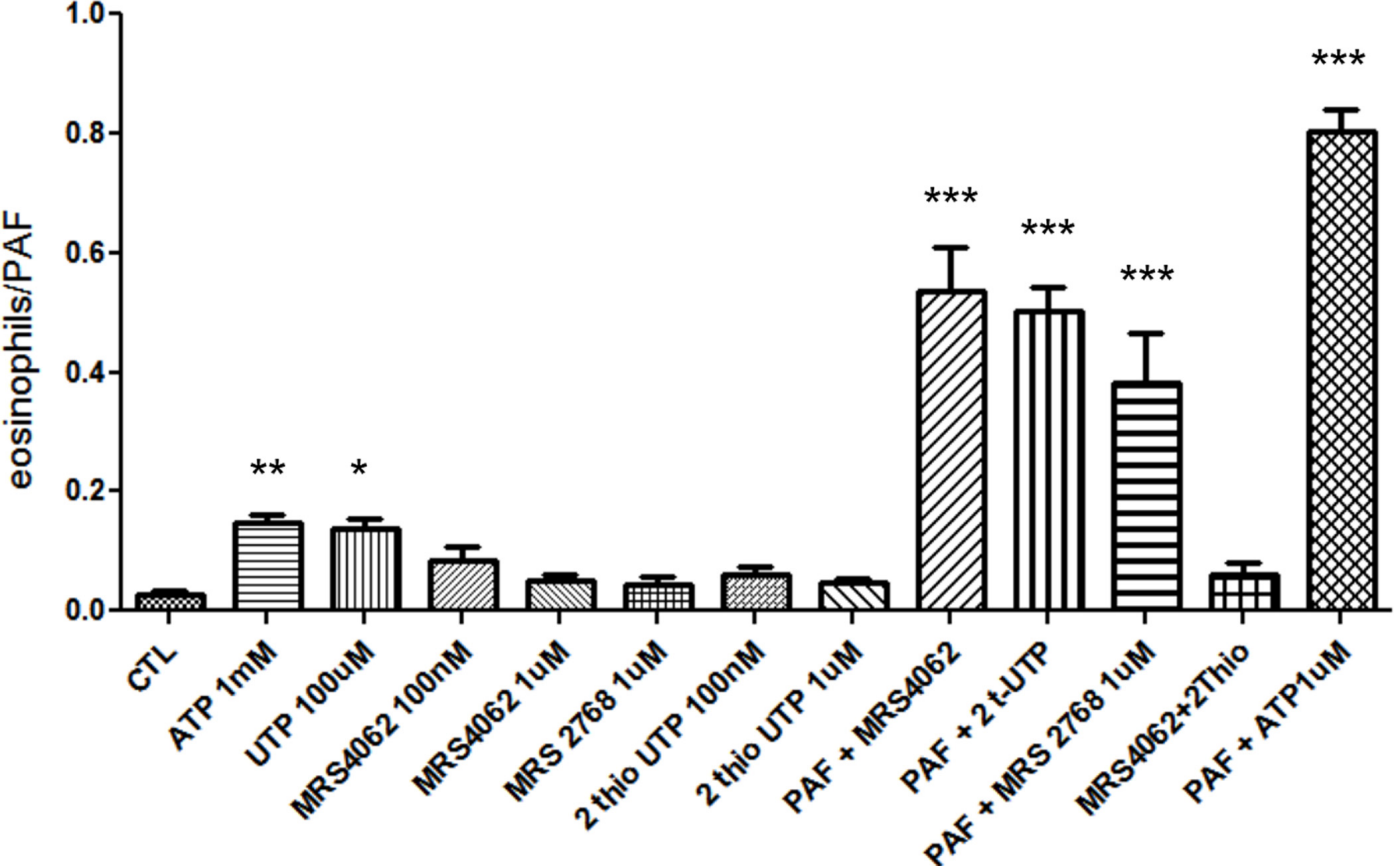
Assessment of rat eosinophil chemotaxis induced by selective P2Y nucleotides using transwell chamber. Transwell chamber has two compartments separated by a 3mm membrane. The nucleotides were added to the lower compartment to induce migration of eosinophils that were in the bottom compartment. After 1h of incubation the cells that migrated were counted by hemocytometer. PAFF was used was a positive control.

Chemotaxis assays using these selective P2Y receptor agonists on rat eosinophils demonstrated absence of effect for all agonists tested at 100 nM and 1 μM for 1 h (Fig 6A).

**Fig 7:**
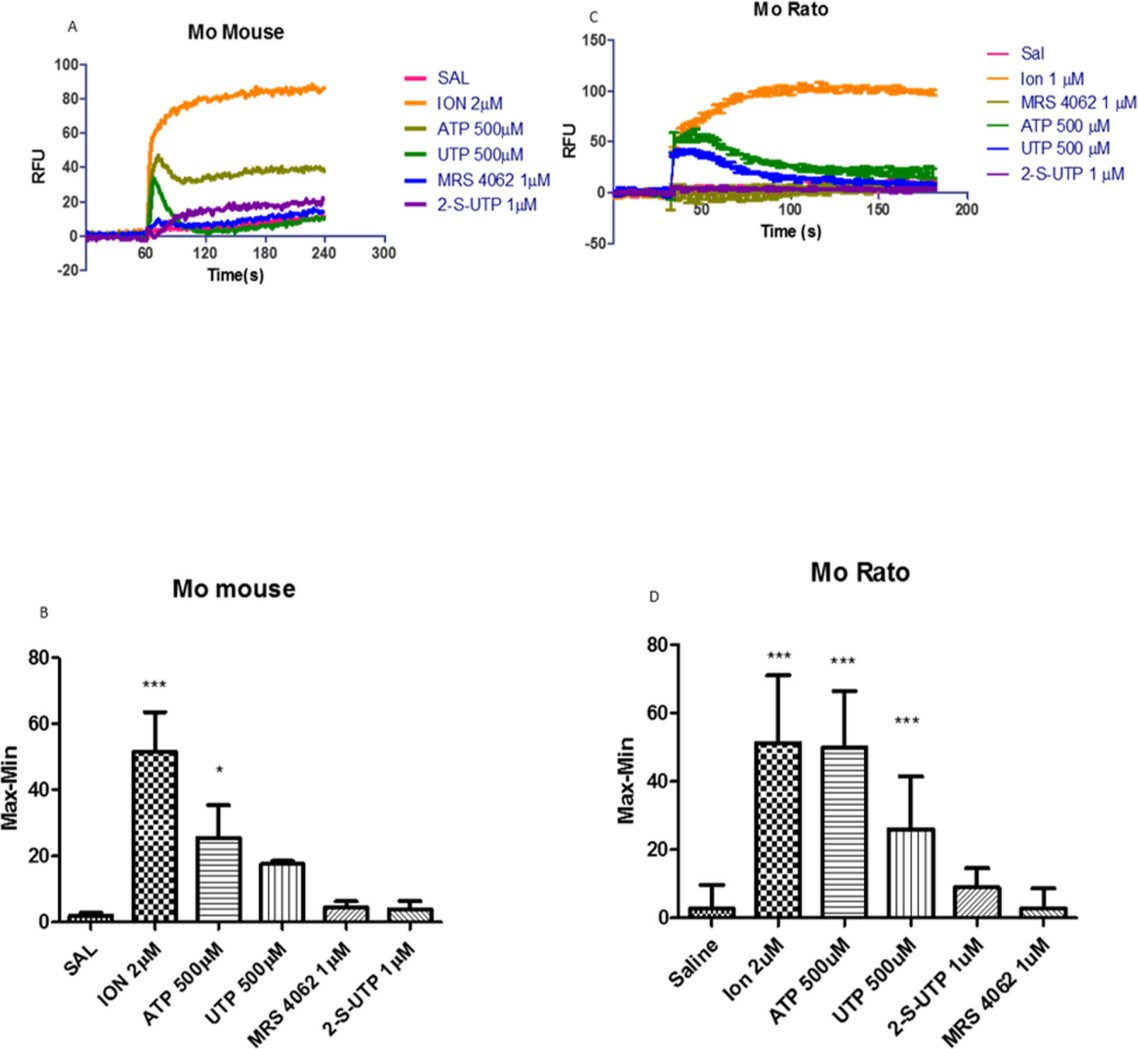
Analysis of calcium transients activated by selective P2Y nucleotides. Rat eosinophils were incubated at 37°C. 2 μM ionomycin was used as positive control. A- 500 μM ATP, 500 μM UTP, 1 μM MRS 4062 and 1 μM 2-S-UTP application on mouse peritoneal macrophages at 37°C for 3 minutes. All recordings were performed at distinct wells. B- Bar graph representation of 2–3 independent experiments. C- 500 μM ATP, 500 μM UTP, 1 μM MRS 4062 and 1 μM 2-S-UTP application on rat peritoneal macrophages at 37°C for 3 minutes. All recordings were performed at distinct wells. D- Bar graph representation of 2–3 independent experiments. *** p<0.01 or * p<0.05 when baseline (saline) was compared to other treatment.

Since these agonists are selective to humans, we tested mice cells response with classical functional P2Y receptor activity assay by measuring calcium transients. We used primary mice peritoneal macrophages, where P2Y receptor already were described [35] and primary rat peritoneal macrophage, where some P2 subtypes, such as P2Y2 receptor, are expressed and functional [36]. Mice peritoneal macrophages stimulated with 500 μM ATP and 500 μM UTP produced an equipotent response (Fig 7A). MRS 4062 and 2-S-UTP did not evoke calcium transients after the treatment with 1 μM of both agonists (Fig 7A and 7B).

Rat peritoneal macrophages treated with 500 μM ATP and 500 μM UTP also produced an equipotent calcium mobilization (Fig 7C and 7D). MRS 4062 and 2-S-UTP also did not induced intracellular calcium variation after the treatment with 1 μM (Fig 7C and 7D). MRS 2768 also applied in the concentration of 1 μM, showed no effect in both cell types (data not shown).

## Discussion

It has long been known that ATP and other nucleotides work as extracellular signaling molecules for intercellular communication in a great variety of cells and tissues, including the immune system [37]. It is also now established that nucleotides act through a complex cellular machinery associated with specific receptors for these nucleotides called purinergic receptors type, P2 receptors. However, the role of P2R in eosinophils in regard to health and disease is little explored. In the present study we have demonstrated that P2X and P2Y receptors are present and functional in rat eosinophils, and that moreover these receptors may play a role modulating migratory responses of these cells in pathological states.

By employing patch clamping whole cell recording we have showed that rat eosinophils have functional P2X receptors. Based on agonist rank potency observed for our results in comparison to rank described in [29], electrophysiological experiments to record P2X receptor activity suggested an essential participation of P2X_2_ receptors. However, the lack of selective P2X_2_ receptor agonist or antagonists impairs this confirmation. P2X_1_ and P2X_3_ receptors may be present. In our records, the fast activation and desensitization currents induced by α, β-me ATP—characteristic of these receptors–recorded a τ similar to P2X_3_ opening that is less than 1s according to Jarvis and Khakh [31]. P2X_4_ and P2X_7_ receptors probably confer a very low contribution (Fig 1A).

We confirmed the presence of P2X_2_, P2X_4_ and P2X_7_ by immunofluorescense assays, as well as P2X_1_. Our data corroborates previous finding by Ferrari and cols (2000) that have reported the presence of the same receptors on human eosinophils with exception of the P2X_2_. Conversely, Mohanty and cols (2001) showed no expression of P2X_7_ in resting eosinophils unless after overnight activation with IFN-γ[4]. In contrast, we have detected the presence of P2X_7_, although we did not detect pore formation by permeabilization assays (data not shown), and observed only a small electrophysiological recording induced by BzATP, when compared to ATP. It is possible that activation of eosinophils with LPS or IFN-γ is required to activate P2X_7_ pores.

Eosinophils were shown to have functional P2Y receptors by the analysis of cytosolic calcium increase after exposure to various analogs of ATP. The results indicate the expression of at least four members of this family: P2Y_2_, P2Y_4_, P2Y_6_ and P2Y_11_. The expression of P2Y_2_ and P2Y_4_ was confirmed by immunofluorescence assays, which also showed the presence of P2Y_1_. The presence of P2Y_11_ receptors is inferred due to calcium transients triggered by ATP and 2meATP, since both are known to act through these receptors [8].

As demonstrated in other studies involving immune cells and human eosinophils [12], we found a nucleotide influence over migration of rat eosinophils. As expected, we have shown that ATP and its analogues were able to induce chemotaxis in eosinophils, yet what was not expected was the indication of P2Y_2_ receptor as the possible essential receptor involved in chemotaxis. This implication arises from the potency order of the nucleotides, being UTP equipotent to ATP, which is a general characteristic of the P2Y_2_ receptors. Interestingly, Muller and cols (2010) showed that P2Y_2_ is a potent inhibitor of chemotaxis in dendritic cells and eosinophils in a knockout mice model [38]. Moreover, Vanderstocken and cols suggested a downregulation of VCAM-1 in P2Y_2_ knockout mice, however neutrophil and macrophage counts in BALF were not altered indicating the participation of others P2 subtypes in migration[39]. Due to UTP effect, we cannot rule out P2Y_4_ and P2Y_6_ participation.

P2X_2_ is another receptor theoretically associated to eosinophil chemotaxis given the equipotent ATP and ATPγS effect and chemotaxis inhibition by PPADS and Suramin (Fig 3A).

Corroborating with these data, we successfully used the pleurisy model in rats to test if in vitro effects of nucleotides on migration could be replicated in vivo. Our data suggest that P2 receptors are participate in the accumulation of eosinophils into the inflammation site, since Suramin almost abolished the presence of eosinophils in the pleural effluent. Similar findings were reported by Idzko and cols (2007) using Suramin and apyrase, an ATPase enzyme, as inhibitors of purinergic receptors [40]. Moreover, reactive blue 2 (RB2) did not block the migration of cells, indicating the P2Y_2_ as the main receptor in migration of murine eosinophils according to previously published data [38]. We did not indicate any P2X receptor since P2X blockers, such as, TNP-ATP, NF023 (data not shown) had no effect in migration of both leukocytes and eosinophils.

As results from eosinophil chemotaxis and pleurisy experiments indicate a primordial P2Y_2_ receptor action, we tested selective human P2Y_2_ and human P2Y_4_ agonists. The P2Y_2_ receptor selective agonists MRS 2768 (data not shown) and 2-S-UTP, and the P2Y_4_ receptor selective agonist MRS 4062 did not evoke eosinophils chemotaxis (Fig 7A) or intracellular calcium mobilization (Fig 7B). This lack of activity may be due to species-specific agonists as a similar profile occurred in rat podocytes [41]. However, in the absence of selective agonists to rat P2Y receptors, agonist rank potency indicate P2Y2 receptor as the key receptor associated with isolated rat eosinophil chemotaxis in vitro and rat pleural migration in vivo.

Taken together these results showed that rat eosinophils express P2R and that these receptors mediate eosinophil migration when activated by extracellular nucleotides. Additionally, these data adds to the similarity observed between rat and human asthma characteristics, which indicates that the study of asthma in rats may represent a reliable and less expensive alternative to uncover mechanisms and test new drugs aimed to treat this disease.
